# Rapid weathering procedure to assess the resistance of exterior renders and facade paints against microbial growth

**DOI:** 10.1016/j.mex.2026.104224

**Published:** 2026-10-30

**Authors:** Christian R. Scherer, Wolfgang K. Hofbauer, Nicole Krueger, Outi Ilvonen

**Affiliations:** a Fraunhofer Institute for Building Physics IBP, Fraunhoferstr. 10, Valley, 83626, Germany; b Umweltbundesamt, Woerlitzer Platz 1, Dessau-Rosslau, 06844, Germany

**Keywords:** Microbial growth, Mold, Algae, Rapid weathering, Facade coating

## Abstract

Facade coatings used in external thermal insulation composite systems (ETICS) are commonly protected against microbial growth by biocides that can leach into the environment. To support the development of biocide‑free products, we developed a rapid weathering method that enables assessment of the resistance of facade coatings to microbial growth. ETICS specimens are mounted in a climate chamber and exposed for 100 ± 5 days to a controlled climate simulating an average Central European autumn day. Surface temperatures of the specimens are actively regulated to fall below the dew point for 8 ± 1 h per day, generating condensation that promotes microbial growth. Natural colonization is simulated by regular inoculation with a defined suspension of algae and mold. Microbial development is documented throughout the test and quantified using a 10-level rating scale that captures both growth onset and intensity. Application of the method to a range of ETICS compositions demonstrated high discriminatory power and good reproducibility. The procedure is proposed for rapid, laboratory-based comparison of biocide-free ETICS concerning their resistance to facade contamination by microorganisms.

## Graphical abstract

Evaluation of resistance of biocide-free facade coatings to microbial growth using the 100-day rapid weathering test.

**Figure:**
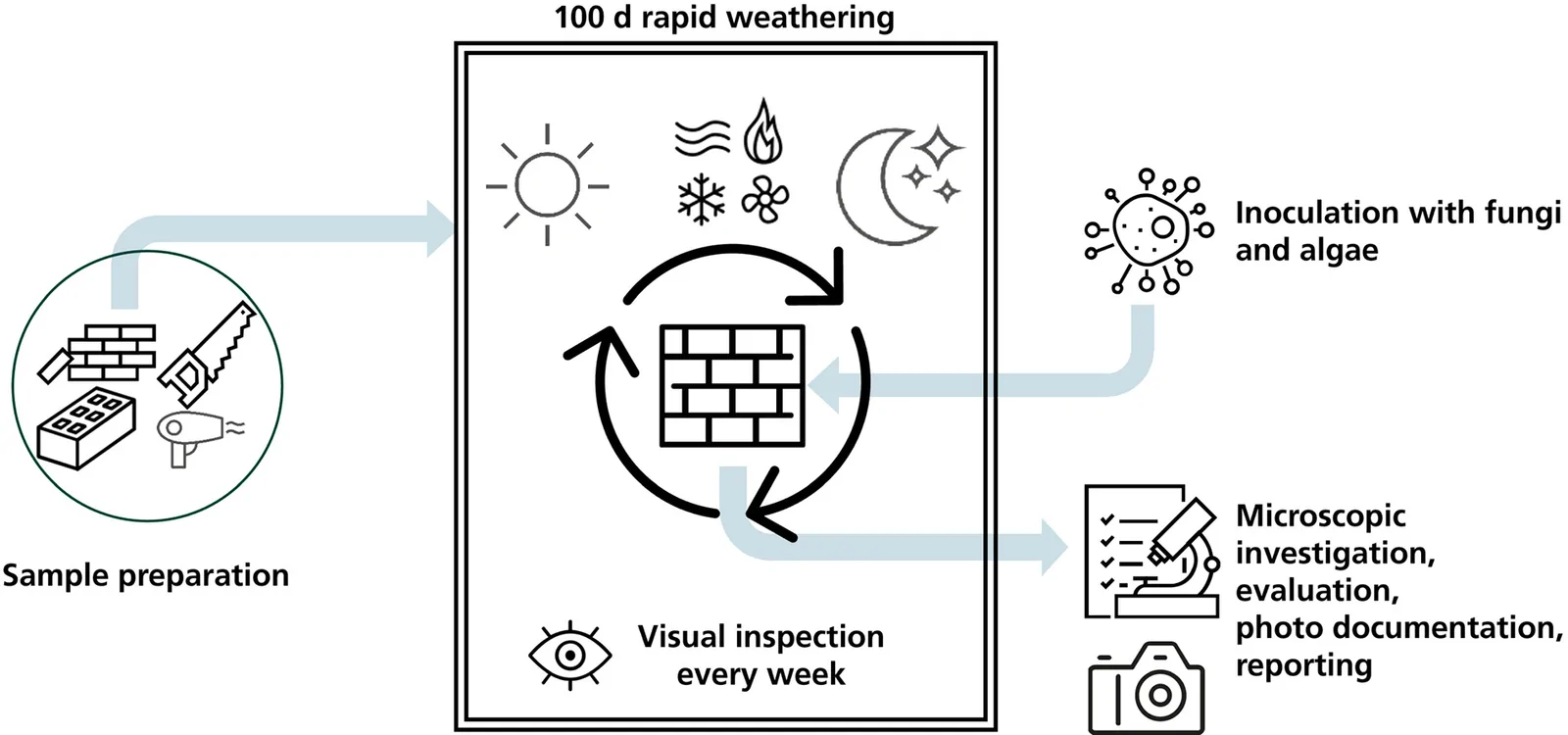

## Specifications table

**Table:** 

**Subject area**	Environmental Science
**More specific subject area**	Resistance of façade coatings and external thermal insulation composite systems (ETICS) against microbial growth; hygrothermal test methods
**Name of your method**	Rapid weathering procedure to assess the resistance of exterior renders and facade paints against microbial growth
**Name and reference of original method**	Rapid weathering system and protocol as developed at Fraunhofer Institute for Building Physics IBP [[1], [2], [3]] and published in Krueger et al., “Resilience of biocide-free ETICS to microbiological growth in an accelerated weathering test”, Building and Environment 244 (2023) 110737 [4].
**Resource availability**	The rapid weathering test facility available at Fraunhofer IBP (Valley, Germany) was developed by Fraunhofer IBP and manufactured by Streit TGA (Streit-TGA GmbH & Co. KG, Holzkirchen, Germany [5]). Test fungi available from the German Collection of Microorganisms and Cell Cultures (DSMZ) [6]. Test algae available from the Culture Collection of Algae at Göttingen University (SAG) [7] and from Fraunhofer IBP. Media recipes (modified Bold’s Basal Medium [8]) and climatic set points are provided in the main text.

## Background

Paste-like renders and facade paints are often treated with biocidal active ingredients to protect them against microbial growth. Environmental labels such as the Blue Angel or the Austrian Ecolabel certify biocide-free thermal insulation composite systems (ETICS) [9] and facade paints [10] but require proof that these systems are resistant to microbial growth. Manufacturers are faced with the challenge of having to prove the resistance of their biocide-free systems to microbial growth. To date, no standardized, widely accepted laboratory method exists that can reliably assess the durability of biocide-free exterior plasters and facade paints. Manufacturers can demonstrate the durability of their products based on their own field tests or proven real-world applications. However, the test specimens must have been installed, or the real-life objects must have been erected at least three years previously, inspected by a trained person, and sufficiently detailed weather data must be available for the respective location. In addition, it must be ensured that the locations did not experience any unusual climatic conditions (e.g., particularly humid or particularly dry) during the observation period. A high risk of infection, e.g., due to vegetation close to the building, must also be ruled out. One alternative, which is also mentioned in the award criteria for the Blue Angel, is a rapid weathering test that covers all the parameters that are decisive for microbial growth on facades: the hygrothermal conditions on the surface (available moisture, temperatures favorable to growth), application of fungal spores and algae from the environment and sufficient light for algae growth. Adverse impacts have also to be considered: air flow and driving rain. In the rapid weathering chamber test specimens are exposed to a climate that promotes microbial growth for 100 ± 5 days (approx. 15 weeks), supplemented by regular inoculation of the test specimens with algae and mold that are typically found on facades. Microbial growth in the rapid weathering chamber was always compared to a three-year outdoor trial of the similar specimens (same variants). A test duration of 100 ± 5 was chosen for practical reasons, as a much longer test duration would not necessarily give more information. Depending on the bioreceptivity of the material surface growth would reach a plateau or intensify with time.

## Method details

ETICS test specimens are exposed to climatic conditions in a rapid weathering chamber over a period of 100 days, based on a daily cycle derived from an average autumn day in Valley (Germany, 47.875026°N // 11.726933°E), which is known to promote microbial growth. The method for deriving the test climate from the climate at the Valley site is described in [2]. The programmed daily cycle includes light and dark phases as well as varying air temperatures and humidity levels, resulting in a period during which the surface temperature of the test specimens falls below the dew point. The surface temperature is lowered below the dew point temperature by the temperature-controlled rear panel of the rapid weathering chamber on which the test specimens are mounted. A surface temperature below the dew point leads to condensation on the surface of the test specimens and thus to the formation of a moisture film, which is essential for microbial growth. The natural deposition of mold spores and algae, as occurs on buildings, is simulated by regularly treating the test specimen surfaces with a suspension containing spores from four mold species and four algae species, repeated at two-week intervals. Light intensity varies throughout the day. Both the light intensity and the emission spectrum of the light sources used promote algae growth. Microbial growth is assessed once a week. A driving rain event is also simulated once a week by spraying the test specimens with demineralized, chlorine-free water (10 l/m²). Once the rapid weathering test is complete, the test specimens are removed from the rapid weathering chamber. The microbial growth is examined under a stereomicroscope, photographed, and finally evaluated.

### Technical requirements

The basic requirements for a rapid weathering facility used to conduct the test described are.

• Rapid weathering chamber made of a material that is inert to common disinfectants and can be sterilized with hot air (120°C).
• With temperature-programmable positions for.
  ○ 5 test specimens (4 x inoculated, 1 x uninoculated) per sample to be tested.
  ○ 4 positive controls.
  ○ Drainage plates.
• Capability for visual inspection.
• Programmable relative humidity and air temperature.
• Programmable airflow velocity.
• Programmable illumination.
• Controllable irrigation system.
• Direct drainage of wastewater into a drain.
• Programmable humidity, temperature, lighting, and spray system

### Rapid weathering facility

The rapid weathering system consists of a rapid weathering chamber, a supply unit for temperature-controlled and humidified air, a cooling system and a heating coil for temperature control of the rear panel, a lighting and irrigation system, and an embedded PC for controlling and regulating the climate and recording the sensor data. The facility offers space for 64 test specimens (12.5 cm x 12.5 cm each). The space required for the system without the chiller is 2.5 m x 4.5 m.

The following infrastructure must be provided for a system of this size:

• - Three-phase (400 V) power supply.
• - Computer network connection (RJ45 recommended).
• - Purified compressed air (activated carbon and particulate filters).
• - Unchlorinated drinking water supply with desalination cartridge.
• - Connection to a chiller for cooling.
• - Option to discharge the water drained from the system directly into a drain.

The rapid weathering chamber (Fig. 1) is made of stainless steel (V2A). The inner surfaces are resistant to cleaning with commercially available disinfectants (e.g. 2-propanol) and disinfection with hot air (at least 110 °C for 4 h). The temperature of the internal rear panel (2.98 m²), on which the test specimens are mounted, can be controlled and allows to adjust the temperature at the surface of the test specimens so that it is above or below the dew point. To control the temperature of the rear panel, it is equipped with pipes through which the tempered cooling medium flows. The rear panel is heated with an adjustable electric heating coil. The control and design of the cooling and heating systems allow for the execution of temperature profiles. The temperature of the rear panel serves as a target variable.

**Fig. 1:**
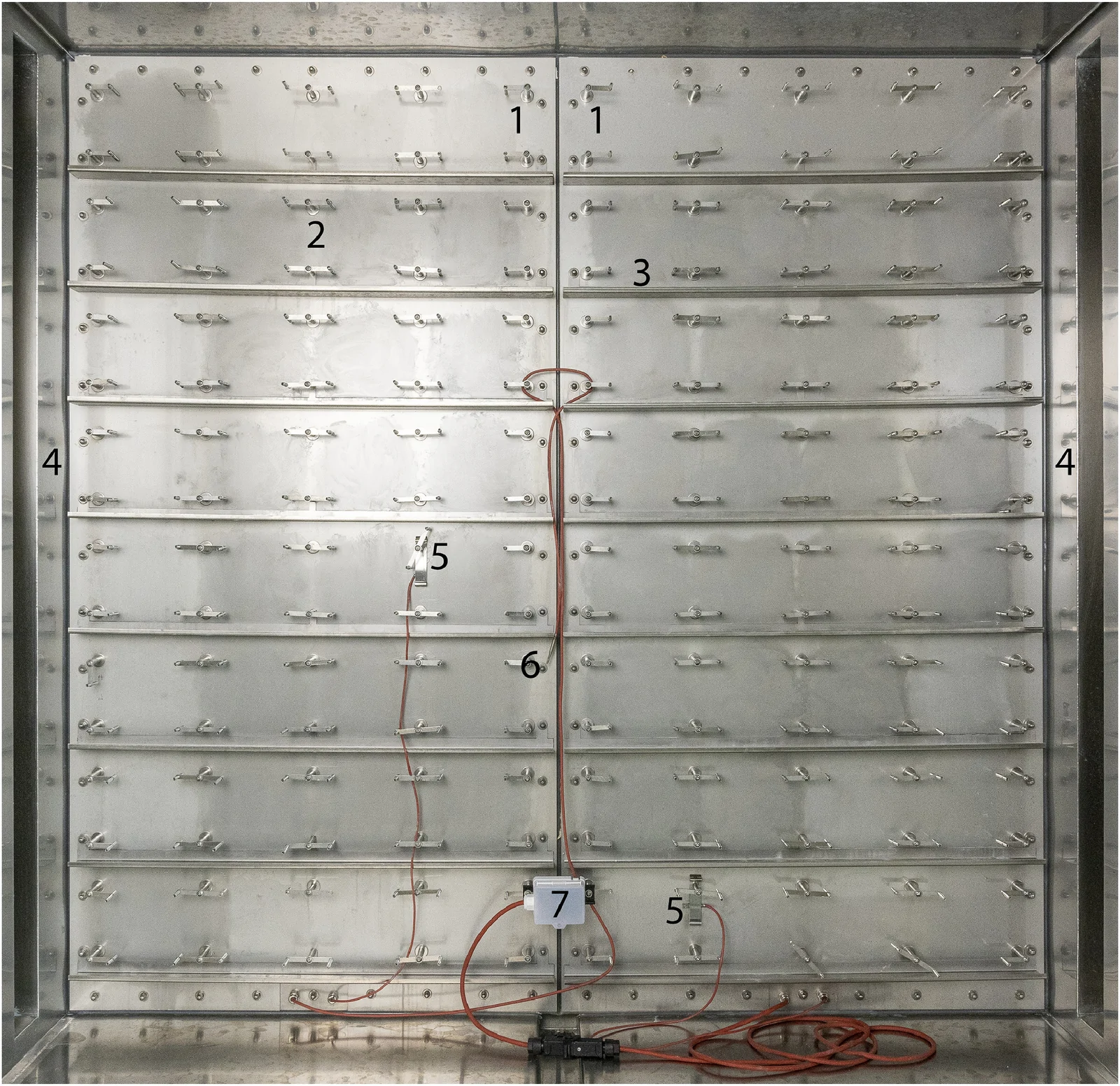
Interior of the rapid weathering chamber: temperature-controlled rear panel (1) with screw bolts (2), drainage plates (3), supply and exhaust air openings (4), temperature (5,6) and illumination sensors (7).

For rapid weathering facilities with a lower sample capacity, it might be also possible to control the surface temperature of the test specimens using Peltier elements.

The test specimens are attached to the rear panel (1) with four screw bolts (Fig. 2) so that they are in full contact. The holders for the test specimens are attached to the rear panel in such a way that the free area of a test specimen not affected by edge effects on which the microbial growth is assessed is at least 10 cm x 10 cm. Drainage plates (3) are installed between the rows of test specimens to prevent water running off one specimen from contaminating the one below. Large-format supply and exhaust air openings (4) are on the side walls. Two temperature sensors (5) for measuring the surface temperature are attached to the rear panel (1). A third one (6) is used to measure the air temperature; it is not fixed and can be placed anywhere (e.g. PT100 1/3 DIN, 4-wire technology, otom® Group GmbH, Braeunlingen, Germany). The illumination sensor (e.g. Li65+ A, Thermokon Sensortechnik GmbH, Mittenaar-Offenbach, Germany) is mounted at the lower center (7). The accuracy of the temperature sensors is 0.1 K, while that of the light sensor is typically 5% of the measurement range.

**Fig. 2:**
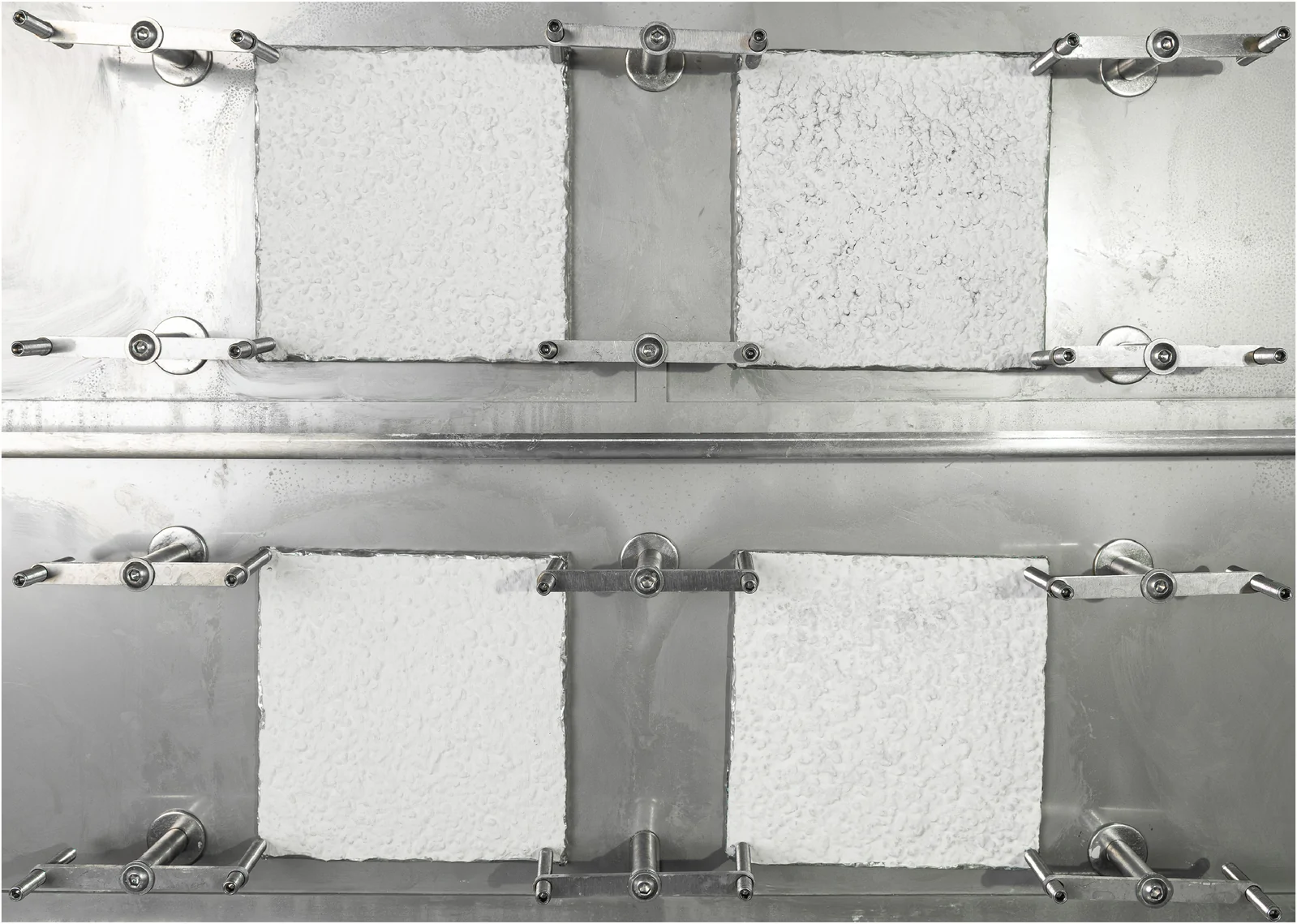
Test specimens (12.5 cm x 12.5 cm) mounted on the rear panel of the rapid weathering chamber. The free area of a test specimen is at least 10 cm x 10 cm. Between the rows of test specimens is a drainage plate that prevents water from contaminating one test specimen from another.

### Air conditioning

Air flow is necessary so that the surface of the test specimens can dry again after the dew point temperature has been reached. The rapid weathering system runs in recirculation mode. The supplied, conditioned air is introduced into the rapid weathering chamber over a large area. The openings for the air supply and the exhaust air discharge are arranged opposite each other so that the air flow sweeps over all test specimens evenly. For this purpose, there are slot-shaped air inlet and outlet openings (Fig. 3) in the side walls. The air speed can be adjusted within a range of 0.0 - 2.5 m/s. The variation in air velocity at different locations within the rapid weathering chamber is ±20% of the set value. Combined temperature/humidity sensors (e.g. HydroClip2 HC2A-SH, Rotronic AG, Bassersdorf, Switzerland) for measuring air temperature and relative humidity are installed on both the air supply and exhaust sides. The accuracy is ±0.5% RH and ±0.1 K at 23 °C ± 5 K.

**Fig. 3:**
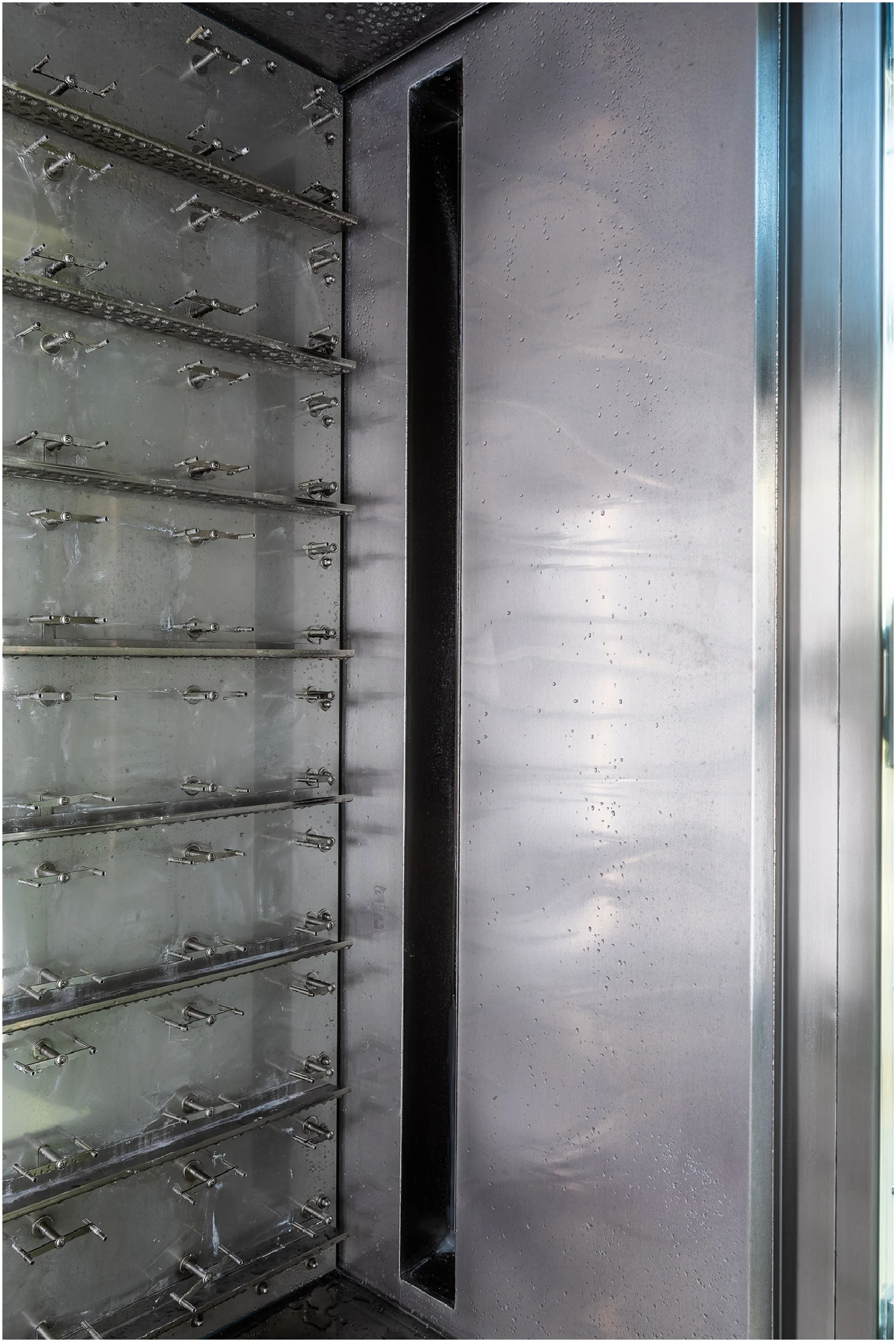
Air inlet in the right-hand side wall.

### Lighting

In contrast to mold, algae need light to grow. Fluorescent tubes and reflectors are installed on the inside of both doors. The fluorescent tubes (e.g. Osram LUMILUX T8 58 W CoolWhite 840, 4000 K, 5200 lm, Osram GmbH, Munich, Germany) provide a radiation spectrum that promotes algae growth, emitting light in the wavelength ranges between 600 and 700 nm and between 400 and 500 nm at a sufficient intensity. The overall illuminance at maximum irradiation is 600 lx on average. The adjustable reflectors are aligned in a way that the test specimens are irradiated as evenly as possible. Deviations over the entire surface do not exceed 20% of the mean value. This means that even in areas with reduced irradiation, the illuminance is still sufficient for algae growth.

### Irrigation (driving rain simulation)

Two spray nozzles (e.g. 1/8GG-SS2.8 W, Spraying Systems Co., Wheaton, USA) are mounted on the inside of each door so that the test specimens attached to the rear panel are evenly exposed to water (Fig. 4). Demineralized chlorine-free water is used for the sprinkling to prevent the formation of a lime layer on the test specimens’ surface. The sprinkling takes place in pulses alternating with intervals without sprinkling (1 min. sprinkling, 9 min. dry interval). This prevents the washing off of any organisms already present on the surfaces of the test specimens. The duration of an irrigation cycle is 1 hour. The amount of precipitation applied to the test specimens during a simulated rainfall event is 10 l/m². The flow rate is adjusted by means of a needle valve installed in the water supply line after the demineralizer cartridge. The setting of the needle valve and thus the flow rate is checked volumetrically. The irrigation intensity at different locations within the rapid weathering chamber should not deviate by >20% from the set value. The water running off the test specimens is drained directly into a wastewater drain.

**Fig. 4:**
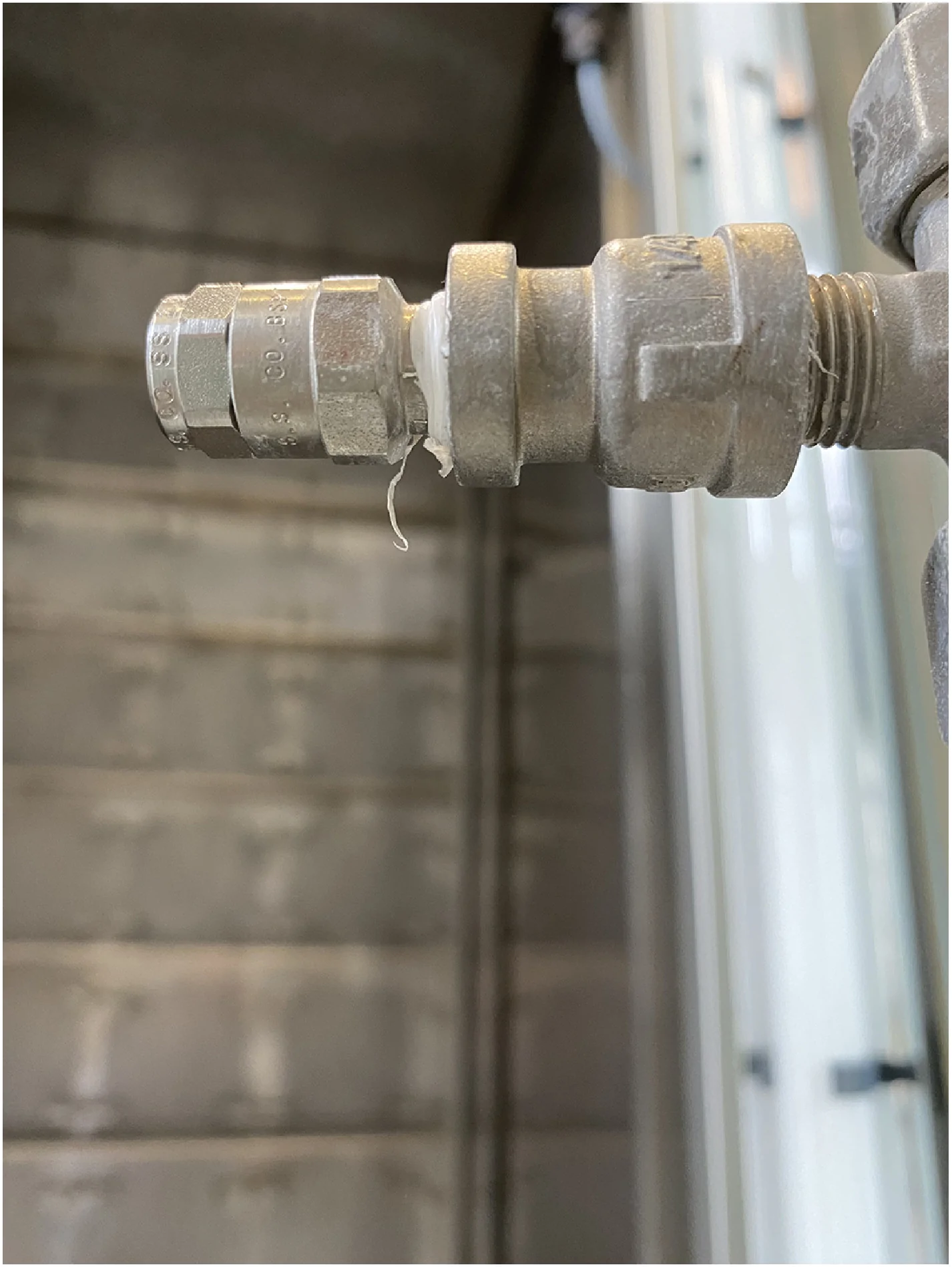
Nozzle mounted inside each door.

### Control and data recording

An embedded PC system (e.g. Beckhoff Automation GmbH & Co. KG, Verl, Germany) with the corresponding IO modules is used to control the system and record the sensor data. The system is operated via a touchscreen built into the switching cabinet. The actual values listed below are recorded over the entire test period:

• Illuminance [lx].
• Air temperature inside the weathering chamber [ °C].
• Humidity [% r. h.] and air temperature [ °C] in the supply and exhaust air duct.
• Temperature of the rear panel [ °C].

The measured values are stored in the system and can be uploaded to a central server. The sensor values are visualized using Grafana (Grafana Labs). Error messages from individual system components are also visualized via Grafana. The system can be monitored, controlled, and restarted after a power failure directly via touch screen, at an office workstation or remotely. Measured values can be exported as csv-file.

### Solutions and media for microbiology

#### Suspension solution

The suspension solution contains 9 g/L NaCl and 1 g/L polysorbate 80 (CAS No 9005–65–6) in demineralized water. Once the components have dissolved completely, the solution is autoclaved. The suspension solution can be stored at room temperature for one week.

Media stock solutions (Table 1) and trace element solutions (Table 2) are needed to prepare Bold’s Basal Medium modified according to Bischoff and Bold [4]. If solutions with the described compositions are commercially available, these can also be used.

**Table 1:** Media stock solutions.

Stock solution no.	Substance	Initial weight [g]
1	NaNo_3_	10.0
2	CaCl_2_ x 2 H_2_O	1.0
3	MgSO_4_ x 7 H_2_O	3.0
4	K_2_HPO_4_ x 3 H_2_O	3.0
5	KH_2_PO_4_	7.0
6	NaCl	1.0

The substances listed are each weighed into a 500 ml laboratory glass bottle and 400 ml of demineralized water is added. The bottles are closed and shaken until the solids have dissolved. The media stock solutions (1 - 6) are stored in a refrigerator (4 - 8 °C). The storage life is six months.

**Table 2:** Trace element solutions.

Trace element solution no.	Substance	Initial weight [g]
1	EDTA KOH	50.0 g 31.0 g
2	H_2_SO_4_ conc. FeSO_4_ x 7 H_2_O	1.0 ml 4.98 g
3	H_3_BO_3_	11.41 g
4	ZnSO_4_ x 7 H_2_O MoO_3_ Co(NO_3_)_2_ x 6 H_2_O MnCl_2_ x 4 H_2_O CuSO_4_ x 5 H_2_O	8.82 g 0.71 g 0.49 g 1.44 g 1.57 g

Laboratory glass bottles (V = 1000 ml) are used to prepare the trace element solutions. After weighing the substances, the laboratory glass bottles are filled up to 1000 ml with demineralized water, closed and shaken until the solids are completely dissolved. The trace element solutions (1–4) are stored in a refrigerator (4 - 8 °C). The storage life is six months.

Bold’s Basal Medium (BBM), modified according to Bischoff & Bold [8]

In a laboratory glass bottle (V = 1000 ml), 940 ml of demineralized water is pre-filled. 10 ml per media stock solution (1 - 6) and 1 ml per trace element solution (1 - 4) are added. The bottle is closed and shaken to mix the solution. The resulting solution is divided equally between two laboratory glass bottles (V = 1000 ml) and sterilized in an autoclave. After cooling under a safety workbench, the bottles are closed and stored in a refrigerator at 4 – 8 °C. The solution is stable under these conditions and can be used for three months.

### Test organisms

Fungi and algae used for the test are known to be able to grow on building facades under different climate conditions, according to studies at several locations in Germany [1,2,11]. A suspension containing all the test organisms listed below (Table 3, Table 4) is applied to determine the resistance of building surfaces to microbial growth.

**Table 3:** Test mold.

Organism	DSM number1)
Alternaria alternata	DSM 62,010
Cladosporium herbarum	DSM 62,121
Epicoccum nigrum (= Epicoccum purpurascens)	DSM 891
Phoma herbarum	DSM 63,184

1) The organisms listed in Table 3 can be obtained from the German Collection of Microorganisms and Cell Cultures (DSMZ) [6] or from other European or international suppliers.

**Table 4:** Test algae.

Organism	SAG number1)
Klebsormidium flaccidum	SAG 335–5
Nostoc commune	SAG 1453–3
Stichococcus bacillaris	SAG 379–1a
Chloroidium ellipsoideum (= Chlorella trebouxioides)	HOKI A39²)

1) Organisms with a SAG number can be obtained from the collection of algae cultures of the University of Göttingen SAG (Germany) [7] or other European or international providers.

²) The applied strain of Chloroidium ellipsoideum (Chlorella trebouxioides) is not offered by the SAG but can be obtained from the Fraunhofer Institute for Building Physics IBP (Valley, Germany).

### Preparation of the suspension with the test organisms

To simulate the exposure of microorganisms to the surface of the test specimens in the rapid weathering chamber, a suspension of mold and algae is applied with a brush. Preparing the suspensions requires a microbiological workstation equipped with a safety cabinet (Class MSW II) and an autoclave. The following work described must be carried out under sterile conditions. All vessels and laboratory equipment that come into contact with the sterilized media or the organisms must be sterilized and stored under sterile conditions until use.

### Cultivation and maintenance of the test mold

A sterilized inoculation loop is used to take a small amount of spores from each fungal organism and spread on a new Petri dish (at least 5 dishes per organism) filled with malt extract agar (MEA). These Petri dishes are sealed with parafilm and incubated in an incubator at 23 °C for at least 3 weeks until dense fungal growth has formed. The test molds are kept according to standard microbiological methods.

### Preparation of the suspension with the test mold

Sterile glass wool (filter floss) is placed in a glass funnel. One MEA Petri with good fungal growth dish is used for each fungal organism to prepare the suspension with the test mold. 10 ml of suspension solution is pipetted into each Petri dish. The spores on the fungal growth are scraped off with a sterile inoculation loop, and the resulting suspension is poured from the Petri dish through the glass wool. Then another 10 ml of suspension solution is pipetted into each Petri dish, carefully swirled and the liquid poured from the Petri dish through the glass wool. The filtrate is centrifuged (2000 rpm, 4 min). The supernatant in the tubes is discarded. 10 ml of suspension solution is added to each tube. After shaking, the suspension obtained is centrifuged again (2000 rpm, 4 min). This procedure is repeated twice. Another 10 ml of suspension solution is added and shaken. The spores of all four fungal organisms shaken out are placed in a sterile glass vessel and covered with aluminum foil.

### Cultivation and maintenance of the test algae

200 ml of Bold’s Basal Medium (BBM) [8] are placed in each of four sterile Erlenmeyer flasks (V = 500 ml). Each algae strain supplied is transferred to an Erlenmeyer flask. The Erlenmeyer flasks are closed with aluminum caps. The flasks with Klebsormidium flaccidum and Nostoc commune are placed on a rotary shaker and shaken at 100 rpm without interruption to prevent the algae from settling. The rotary shaker should be positioned so that it is exposed to daylight but not to direct sunlight. The Erlenmeyer flasks with Stichococcus bacillaris and Chloroidium ellipsoideum are kept in a cooled incubator with lighting (e.g. Philips TL-D 36W/840, 3350 lm) at 15 °C. The lighting should be switched on for 8 h per day.

### Preparation of the suspension with the test algae

Approximately 50 ml is taken from each algae strain and combined in a sterile container. All four algae strains are then each topped up with 50 ml of sterile BBM.

### Preparation of the suspension for inoculation of the test specimens

The combined algae suspension is added to the combined fungal spore suspension. This inoculation suspension contains between 10⁵ and 10⁶ colony-forming units per milliliter and is used for inoculation of the test specimens and the positive references. A suspension is prepared from each test organism. The individual suspensions are counted using a Thoma counting chamber and then combined to form the inoculum suspension.

### Test specimens’ preparation

ETICS are built up on polystyrene insulation boards (EPS or XPS, e.g. 100 cm x 50 cm), consisting of reinforcing plaster, and reinforcing fabric, finishing plaster and, if necessary, paint, according to manufacturer’s instructions. After the ETICS sample has fully cured (28 d), seven test samples (12.5 cm x 12.5 cm) per ETICS variant are cut out (Fig. 5). Five test samples are required for the test, two are to be retained as backup samples. Five test samples per ETICS variant are soaked three times for 24 h in chlorine-free demineralized water (80 l/m²) to remove residues of in-can preservatives. The water is exchanged before each immersion. The test samples from each ETICS are placed in a plastic tray (e.g., 40 cm x 40 cm x 15 cm) filled with 6.25 l of water, ensuring that the insulation material faces upward. When immersing the test samples, care should be taken to ensure that there are no air bubbles under the plaster surface. If the coated side does not dip deep enough into the water, the test samples can be weighed down with inert material (glass, stainless steel). Between waterings, the test samples are dried in a drying cabinet for 24 h at 40 °C in recirculation mode. After the final drying step, the test samples may be stored uncovered in the dark at 23 °C and 50% relative humidity for up to one month until they are installed in the rapid weathering chamber.

**Fig. 5:**
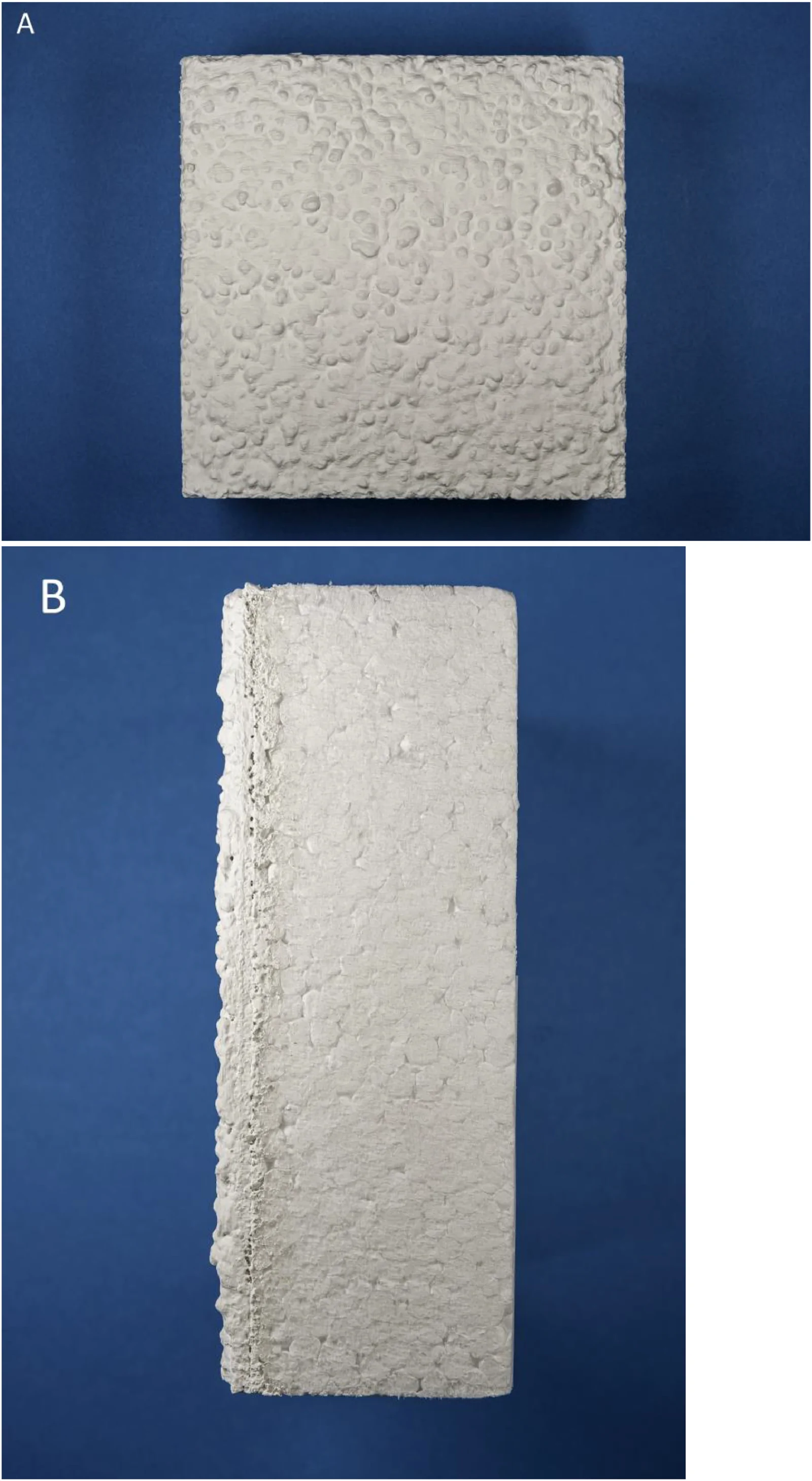

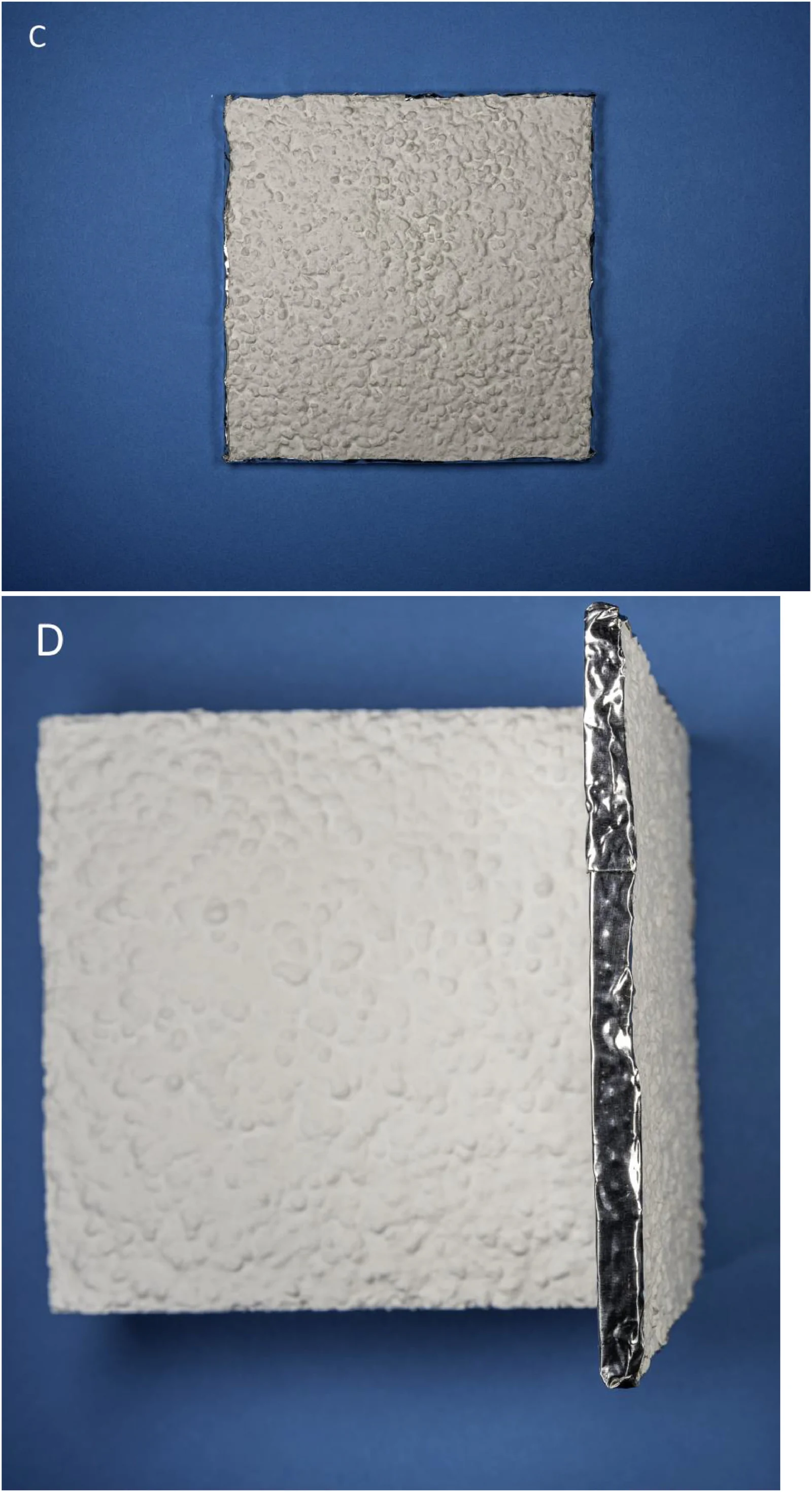
Test specimen: A) ETICS assembly cut to 12.5 cm x 12.5 cm. B) ETICS assembly with EPS insulation board. C, D) ETICS assembly, insulation material removed, edges and rear side sealed with aluminum tape.

Prior to installation the insulation material is completely removed from the coating structure using a cutter knife (Fig. 5). The reinforcing plaster must not be damaged. Then the edges and back of the coating structure are sealed with aluminum adhesive tape. Thermally conductive paste (silicon-free, thermal conductivity 0.5 W/mK @ 25 °C or better, e.g. V6514-D, Assmann WSW components GmbH, Luedenscheid, Germany) is applied thinly (thickness up to max. 1 mm) over the entire back side of the test specimens to even out any unevenness and ensure good and rapid heat transfer between the test specimen and the temperature-controlled rear panel. Removing the insulation layer is necessary to ensure that the required thermal effects can be applied to the surface of the coatings. Although this represents a certain simplification compared with the actual assemblies, it enables technical implementation with a reasonable level of effort. Ultimately, the primary purpose of the method is to compare the top coatings.

### Installation in the rapid weathering chamber

Five test specimens for each ETICS system variant are randomly distributed across the rear panel and affixed with screw bolts. Care must be taken to ensure that the entire surface of the back of the test specimens is in full contact with the temperature-controlled rear panel of the rapid weathering chamber.

### Positive control

The positive controls consist of filter papers that have the same dimensions as the test specimens (Fig. 6). The backside of the positive controls is also taped with aluminum adhesive tape and coated with thermally conductive paste. They are inoculated with organism suspension at the same intervals as the test specimens. This is to check whether a) the light intensity is sufficient to promote algae growth over the entire duration of the test and b) whether the fungal suspension is sufficiently vital.

**Fig. 6:**
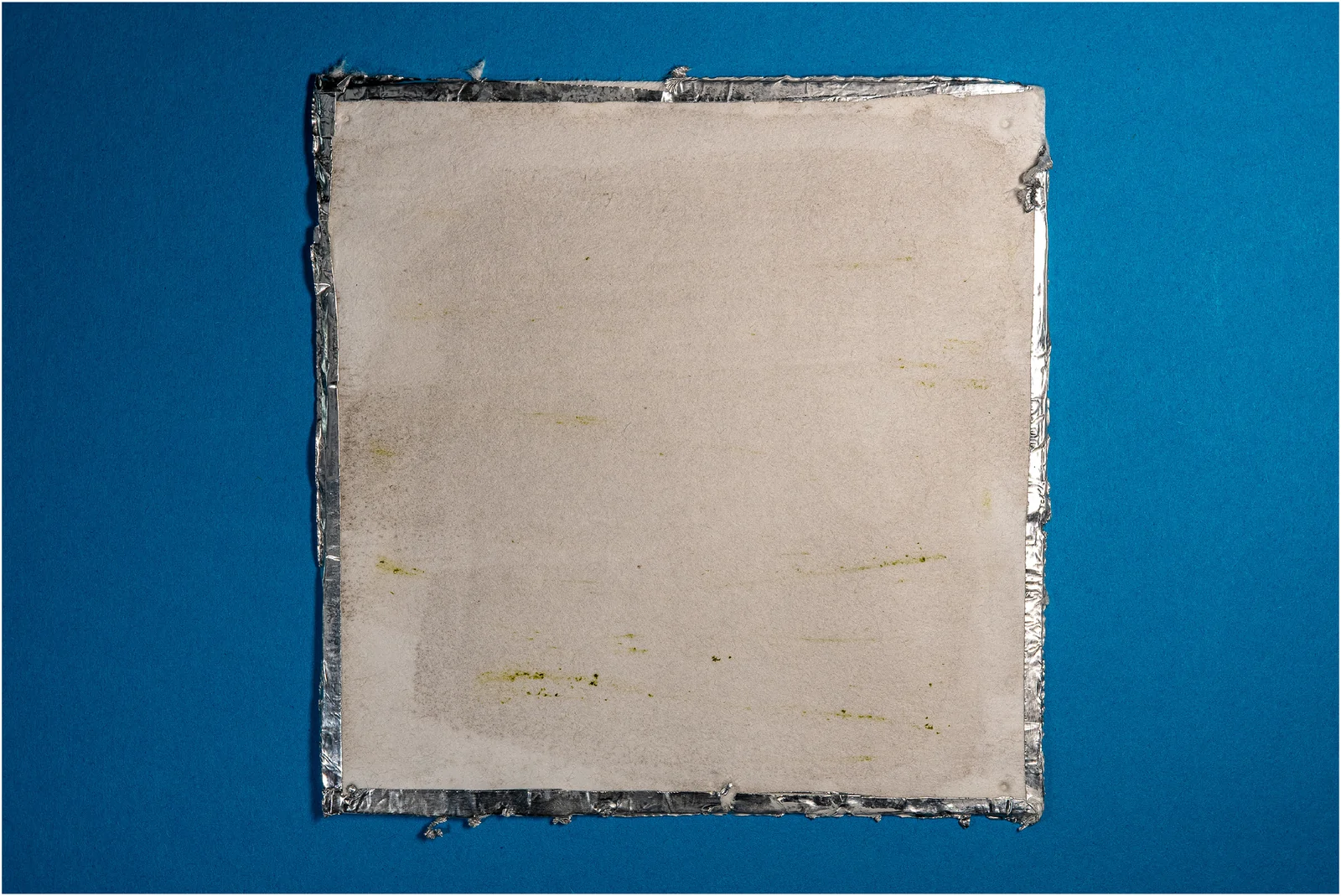
Positive control after 100 days of rapid weathering with microbial growth (grey area = mold, green spots = algae).

### Negative control

One specimen from each sample is not inoculated. This serves as a negative control. Negative control is used to determine whether a sample was already contaminated with microbes before the rapid weathering and whether mold and algae were transferred to neighboring test specimens during irrigation.

### Weathering conditions

The hygrothermal situation on the surface of the test specimens is decisive for microbial growth. In the rapid weathering chamber, the surface temperature of the test specimens falls below the dew point temperature due to cooling of the rear panel for at least 1.5 K for (8 ± 1) hours per test day. The climate parameters for the model climate applied are summarized in Table 5.

**Table 5:** Setpoints in the rapid weathering chamber.

Runtime [h]	Air temperature [ °C]	Temperature of the temperature-controlled rear panel [ °C]	Relative humidity [%]	Light intensity [lx]	Air flow velocity [m/s]	Irrigation [l/m²]
0 - 1	7.8	5.0	91.2	0	0.5	–
1 - 2	7.6	4.7	91.3	0	0.3	–
2 - 3	7.5	4.6	91.4	0	0.3	–
3 - 4	7.4	4.3	91.4	0	0.3	–
4 - 5	7.3	4.7	91.2	0	0.3	–
5 - 6	7.2	4.7	91.4	60	0.3	–
6 - 7	7.0	4.7	91.6	180	0.3	–
7 - 8	6.9	4.7	91.6	180	0.3	–
8 - 9	7.7	6.5	90.7	300	0.3	–
9 - 10	9.2	9.2	86.8	300	0.3	10 1)
10 - 11	10.7	10.8	80.4	600	0.3	–
11 - 12	11.7	11.9	75.7	600	0.5	–
12 - 13	12.5	12.9	72.6	600	0.5	–
13 - 14	13.0	13.5	70.9	600	0.5	–
14 - 15	13.4	13.8	69.3	600	0.2	–
15 - 16	13.7	14.3	68.7	600	0.8	–
16 - 17	13.4	14.2	69.4	300	0.8	–
17 - 18	12.7	13.9	72.9	300	0.8	–
18 - 19	11.5	12.5	79.1	150	0.8	–
19 - 20	10.2	10.6	84.6	0	0.8	–
20 - 21	9.1	9.0	88.3	0	0.5	–
21 - 22	8.4	8.3	90.3	0	0.5	–
22 - 23	8.2	8.0	91.0	0	0.5	–
23 - 24	7.9	7.7	90.9	0	0.5	–

1) Rain simulation takes place once per test week.

The air temperature ranges from 6.9 °C to 13.7 °C, and the temperature of the rear panel ranges from 4.3 °C to 14.3 °C. The minimum relative humidity is 68.7% RH, and the maximum is 91.6% RH. The lighting starts at 60 lx after 5 h and reaches its maximum of approximately 600 lx after 10 h. This level is maintained for 6 h. The daily variations in temperature, relative humidity, and irradiance are shown in Fig. 7. The chosen autumn climate based on real climate conditions in Valley represents a typical but challenging climate for Germany / Central Europe promoting surface growth on building structures.

**Fig. 7:**
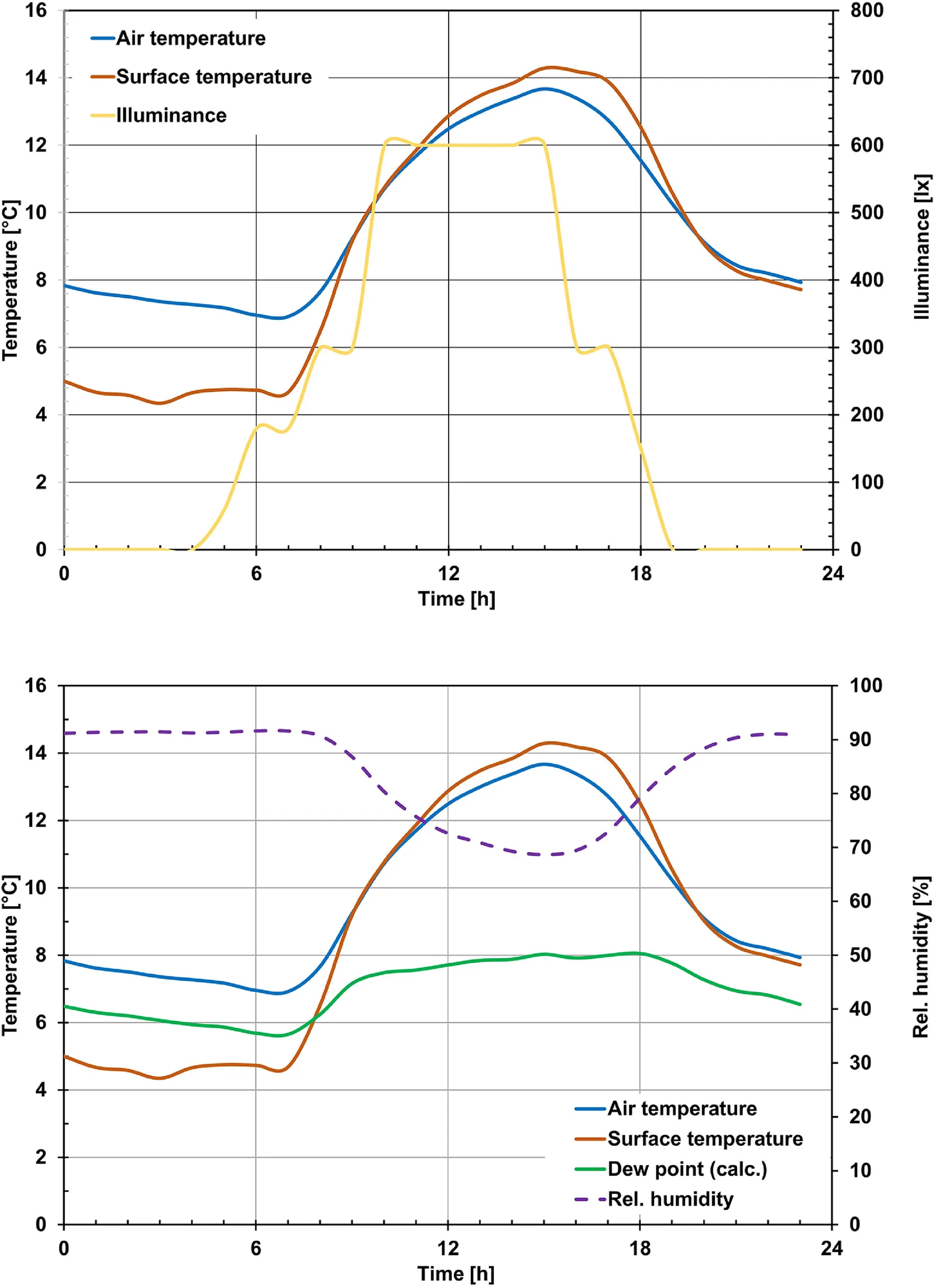
Experimental conditions: Left: Air and surface temperature and illuminance. Right: Air and surface temperature, calculated dew point temperature, and relative humidity.

### Irrigation

Once per test week, the specimens are irrigated with demineralized water. The interval between two spraying cycles is one week. Weekly spraying takes place in intervals. One minute of spraying is followed by a dry phase of 9 min. Overall, a water volume is sprinkled in the rapid weathering chamber that corresponds to a precipitation volume of 10 l/m² of test specimen surface.

### Inoculation

The first inoculation of the specimens and positive references is carried out two days after the first irrigation. The inoculation is repeated five times at two-week intervals. Four of the five test specimens per ETICS are inoculated with the suspension containing mold and algae. The fifth test specimen remains untreated. The combined fungal and algal suspension is thoroughly stirred with an autoclaved brush. The brush is carefully wiped against the inside of the container. Specimens and positive references are evenly coated with the suspension so that the entire surface is wetted. Dripping of the suspension shall be avoided. In this procedure, an estimated 3 mL of the inoculation suspension is applied to a test sample.

### Inspection and documentation during rapid weathering

Fungal growth on the test specimens and on the positive and negative reference samples is visually inspected at weekly intervals by trained personnel. We recommend using a grid template (Fig. 8) to estimate the area covered by growth. The evaluation is performed according to the evaluation scheme (Fig. 9). The intensity of fungal growth is recorded, and each test specimen is photographed. Algal growth usually is not visible to the naked eye during rapid weathering. Abnormalities and changes (e.g. bulging, cupping, cracks, discoloration) in the test specimens are documented. At regular intervals, the trained personnel assess microbial growth both on specimens exposed outdoors and in accelerated weathering tests and compare their results with one another.

**Fig. 8:**
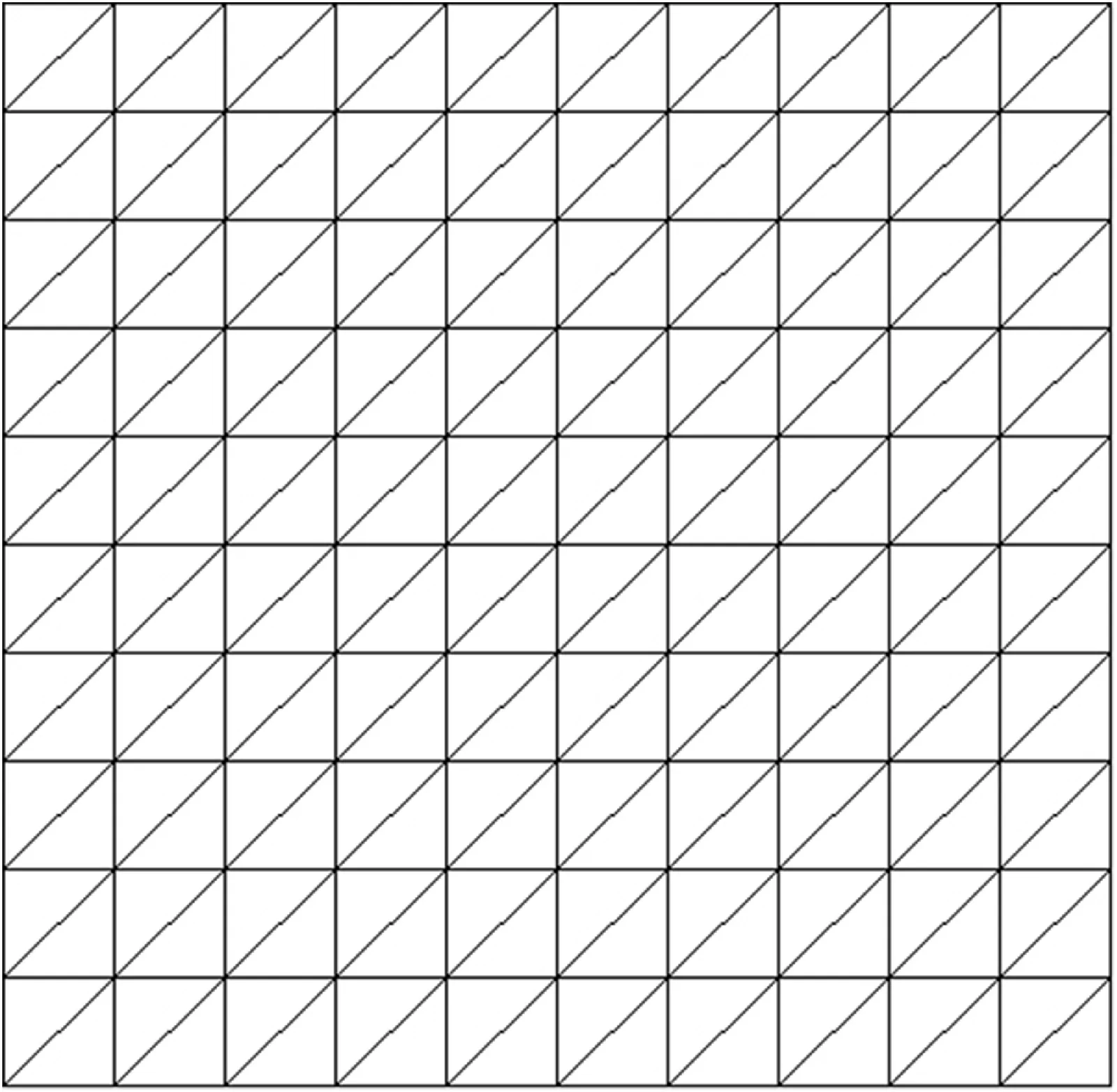
Grid template for visual recording of microbial growth on the test specimens. (schematic).

**Fig. 9:**
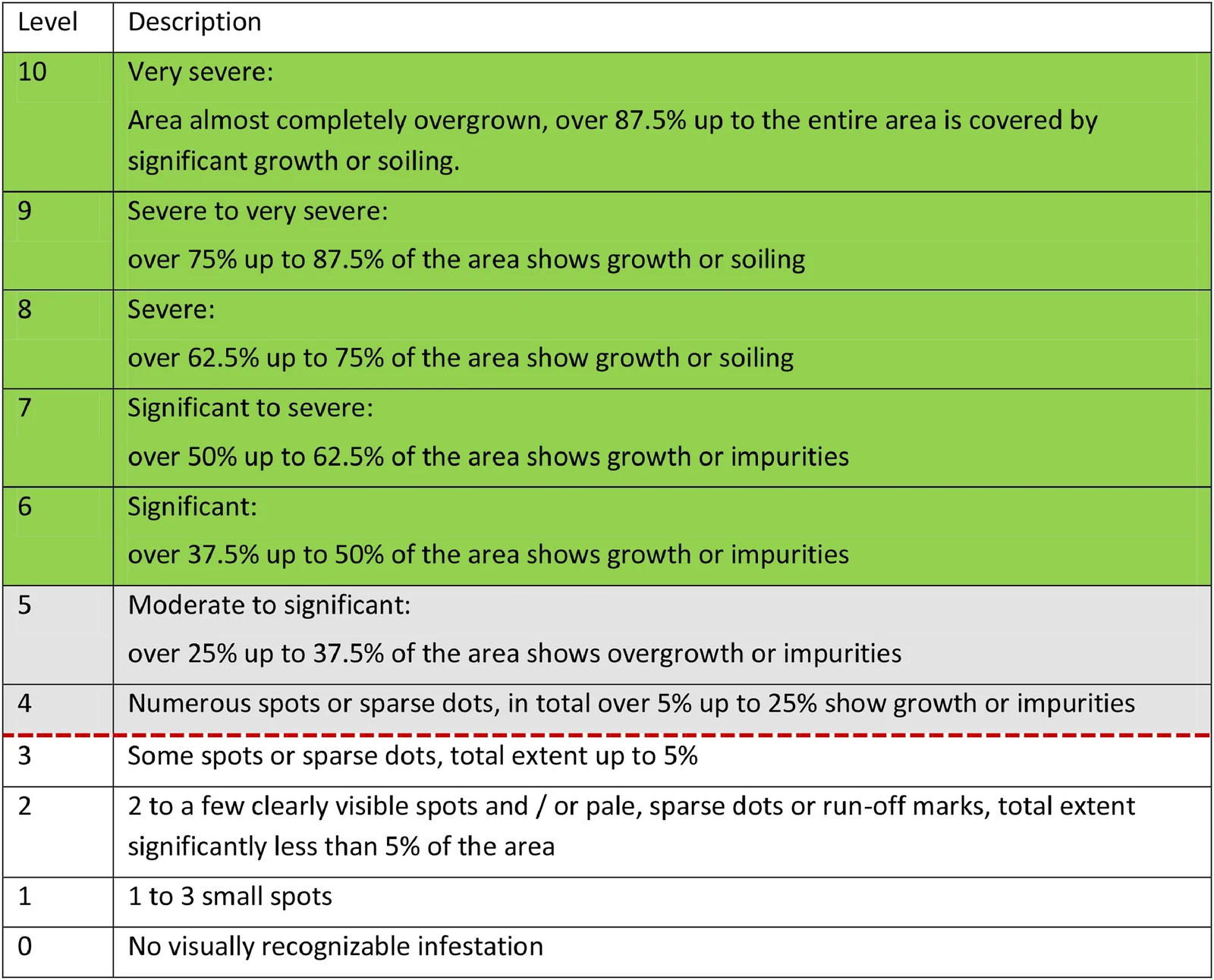
Recording scale for visual assessment of the test specimen growth. Experience has shown that complaints can arise with assessment levels of 3 to 5 or higher.

### Assessment after the end of rapid weathering

Immediately after completion of the rapid weathering, all specimens as well as the positive and negative references are documented photographically. Then the test specimens and references are removed from the rear panel. The remaining heat transfer paste is removed from the test specimens and references without leaving any residue. The specimens and references are stored in Petri dishes or in polyethylene zip lock bags at room temperature in a dark and dry place. The evaluation should be carried out within two days of removal to prevent, for example, the algae from being overgrown by mold.

For evaluation a grid template (Fig. 8) measuring 10 cm x 10 cm is placed on the test specimen’s surface and the areas covered with mold are counted. Fields (1 cm x 1 cm) that are more than half covered are counted as "1″, fields that show growth, but which cover no more than half of the field are counted as "0.5". The values are added up. The numerical value obtained is used for the evaluation according to the assessment scale (Fig. 9). The growth is then examined under a stereo microscope. After 100 days of artificial weathering, some algae growth is not yet visible with the naked eye. Therefore, the algae growth is assessed using a stereomicroscope (magnification 6.5x – 8x, standard illumination). To ensure that the results are comparable, the magnification and illumination should not deviate from the specified settings.

Fungal growth is evaluated according to the evaluation scheme (Fig. 9) again and documented photographically by means of detailed images of the overgrown areas. The photo documentation is part of the test report. Typically, surface contamination on exterior façades consists of interconnected organic and inorganic components. In rare exceptional cases, the contamination or discoloration is purely inorganic. This is not expected with the test method presented. However, to ensure that the scale can also be applied generally in other situations, such as outdoor exposure, the terminology has been broadened with “soiling” and “impurities” [11].

The evaluation scheme consists of four categories describing resilience against mold:

• 0:no visible growth.
• 1 - 2:isolated colonies (< 5% of area).
• 3 - 5:increasing coverage (5 – 37.5% of area), typically associated with first customer complaints.
• 6 - 10:extensive to almost full coverage (> 37.5 – 90% of area), user complaints are very likely.

The assessment of facades in cases of claims and disputes revealed that the first user complaints typically arise in the range of assessment levels 3–5.

Each test specimen is evaluated individually. Then, the four individual values are averaged. The mean values are displayed in a bar graph (Fig. 10). The assessment considers the intensity of the growth (level 0 – 10) or contamination, as well as the time (week 0 – week 15) when growth first becomes visible.

**Fig. 10:**
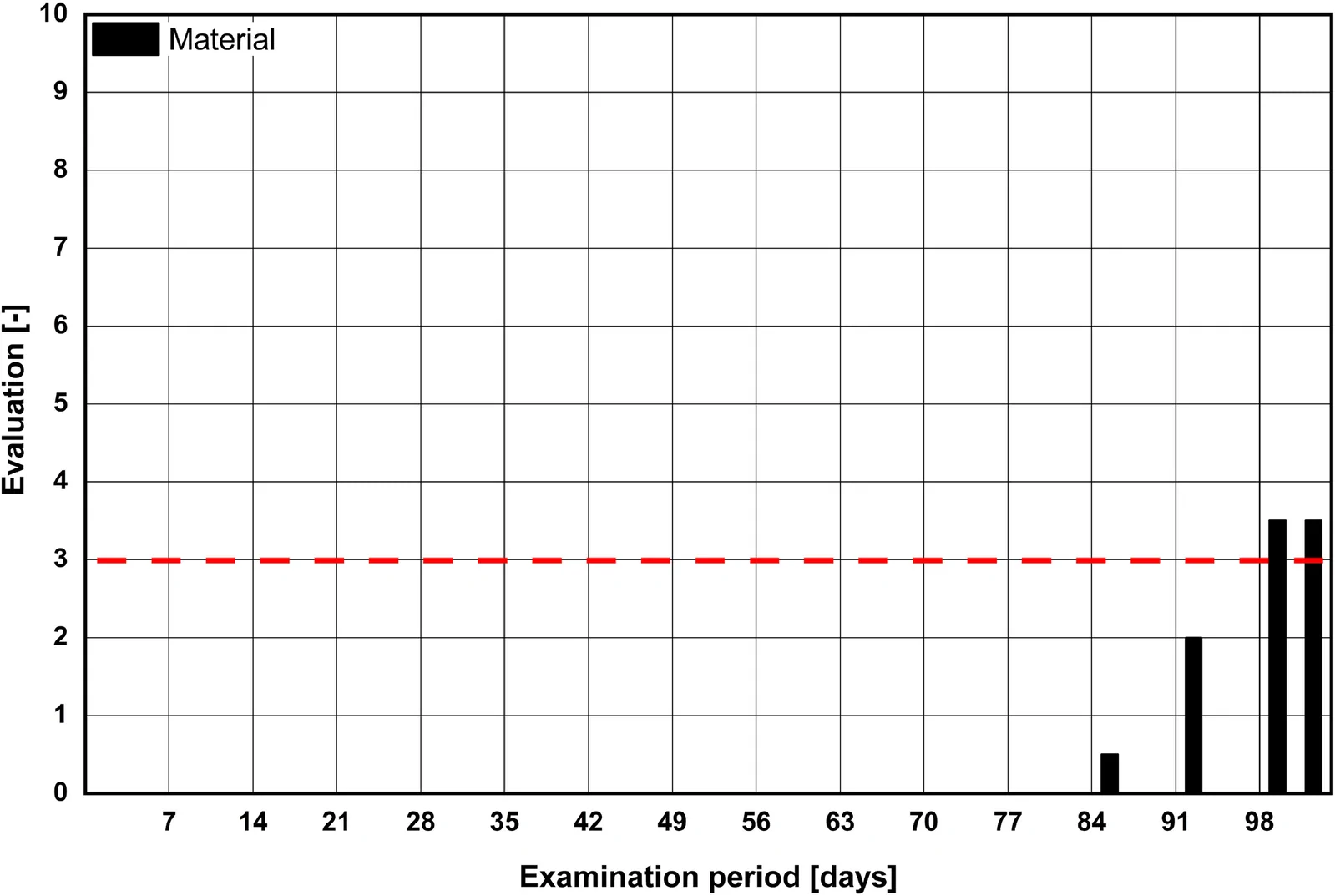
Visualization of the growth development (mold) over time (example.)

To assess the resistance of an ETICS to fungal growth, a rating scale with five levels is used in the Blue Angel award criteria [9] and [10]:

• W1 High: The fungal growth remained below level 3 on the assessment scale over the 100 days
• W2 Good to medium: The fungal growth slightly exceeded level 3 on the assessment scale but only towards the end of the test cycle.
• W3 Medium: The fungal growth exceeded level 3 on the rating scale, but did not appear until relatively late in the test cycle and did not reach a serious extent.
• W4 Medium to low: Clear development of fungal growth
• W5 Low: Significant fungal growth developed relatively quickly and reached a high level by the end of the test cycle.

Algae can usually only form very small colonies within 100 days. Consequently, algal growth can only be classified into three levels:

• A1 High: No algae growth visible.
• A2 Medium: Algae visible only under a stereomicroscope.
• A3 Low: Algae visible to the naked eye at the end of the rapid weathering process.

Based on the extent of microbial growth at the time of inspection, and differentiating between mold and algae, a final assessment of resistance is then conducted. The criteria may vary depending on the applicable award criteria. The Blue Angel award criteria for ETICS [9] and exterior paints [10] require a minimum rating of W3/A2, i.e., moderate resistance against the growth of mold and algae. Currently, the timing of the onset of visible microbial growth and the progression are not taken into account in the assessment. It is possible to further develop the assessment scheme by introducing an additional assessment level for growth that begins late and remains at a low level until the end of the test cycle.

### Quality assurance

The functionality of the system and ensuring that the setpoints are maintained must be guaranteed throughout the entire weathering cycle. For this reason, measures are needed to check the system and for quality assurance.

If the negative reference shows the same or more growth than the inoculated test specimens, the cause must be identified and remedied, and the test must be repeated.

The positive reference helps to ensure that a) the light intensity is sufficient to promote algae growth over the entire duration of the test and b) the fungal suspension is sufficiently vital. If no growth of algae and mold can be seen on the positive control, the cause must be identified and remedied, and the test shall be repeated.

The temperature and humidity sensors, flow sensors and the illumination sensor must be checked for functionality before the start of the investigation. The check is carried out by means of a comparative measurement with externally calibrated sensors. The temperature and humidity sensors must be recalibrated at regular intervals.

To disinfect the system, light sources, lamp holders, reflectors, and illumination sensors shall be removed. The rapid weathering chamber is disinfected at 110 °C for 4 h. After cooling down, the removed components are reinstalled in the chamber and the reflectors are readjusted. The adjustment is done by measuring the illuminance distributed over the rear panel and aligning the reflectors so that the criteria mean value 600 lx and maximum tolerance ± 20% are achieved again.

The illuminance must be checked and documented before and after each examination. If the required illuminance can no longer be achieved, the light sources must be replaced.

The water volume for driving rain simulation must be checked volumetrically and adjusted before the tests start.

### Method validation

The underlying research projects [[1], [2], [3],^9^,^10^] demonstrated the suitability of the rapid weathering method described here in terms of its reliability and discriminatory power for plasters and paints from different manufacturers and with different formulations.

### Reproducibility

For each ETICS, four replicate specimens are inoculated and exposed in parallel under identical climatic conditions. This design allows the repeatability of the method to be quantified based on the distribution of final growth scores within an ETICS and, if available, across repeated test runs. In our validation experiments with biocide-free ETICS described in the related research articles [1,2,4] and in the Blue Angel development project [3], variability within a given ETICS was small compared to the differences observed between systems, indicating that the scoring procedure and the imposed boundary conditions are sufficiently robust for comparative purposes.

### Discriminatory power

The rapid weathering test is designed to quantify differences in the resistance of ETICS or facade paints to mold and algae growth [2,4]. For this purpose, the materials are exposed to a microclimate that promotes growth and to repeated inoculation with representative mold and algae. When applied to biocide-free ETICS with different compositions, the method revealed clearly distinguishable growth patterns and endpoints for systems with varying formulations and surface properties [1,4]. Figs. 11a through 11d show the results of tests conducted on various facade coatings. Resistance was classified according to the requirements of the award criteria for ETICS (DE-UZ 140, [9]) and low-pollutant facade paints (DE-UZ 225, [10]). Fig. 11a, Fig. 11b show the microbiological growth on two materials that meet the Blue Angel requirements. Fig. 11c, Fig. 11d show the microbiological growth on two ETICS that do not meet the requirements.

**Fig. 11a:**
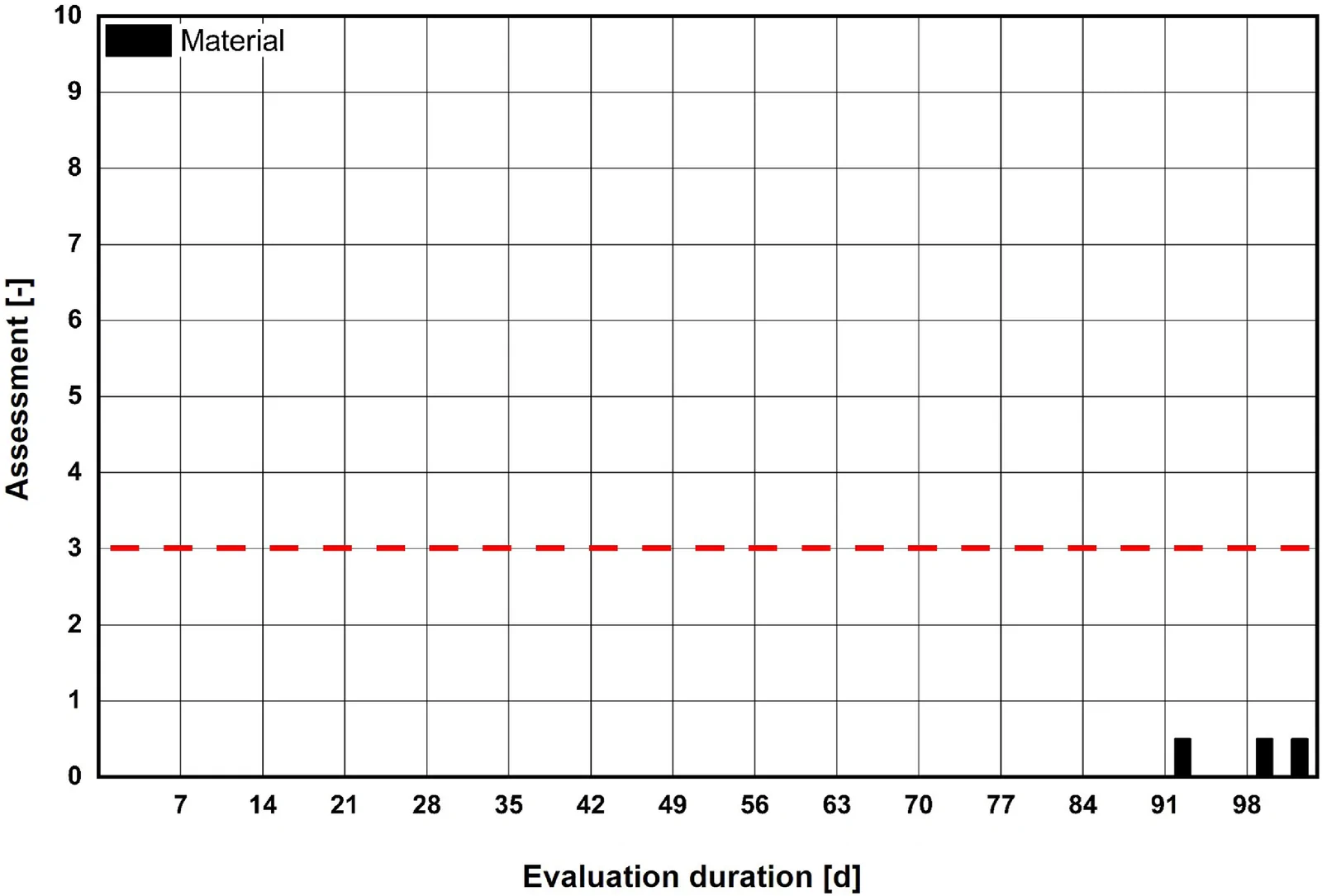
Biological growth first appears in the 14th week of the test. The growth never reaches the critical level of “3.” The facade coating exhibits very good resistance to microbial growth (mold). The facade coating meets the W3/A2 requirements given in [9] and [10].

**Fig. 11b:**
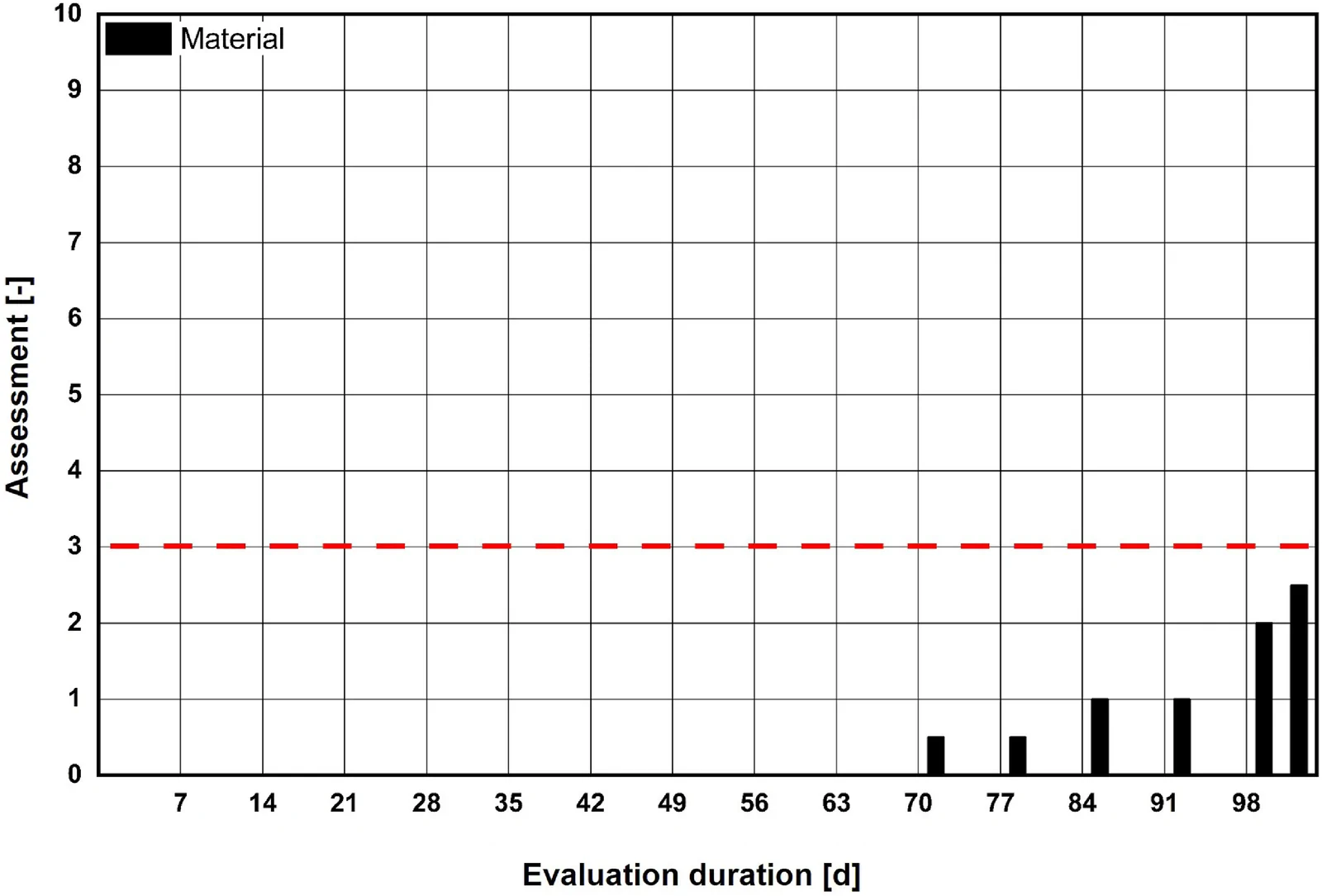
Fungal growth first appears in the 13th week of the test. The fungal growth first exceeds the critical threshold of “3” in the 15th week of the test. The facade coating still exhibits sufficient resistance to microbial growth (mold). The facade coating meets the W3/A2 requirements.

**Fig. 11c:**
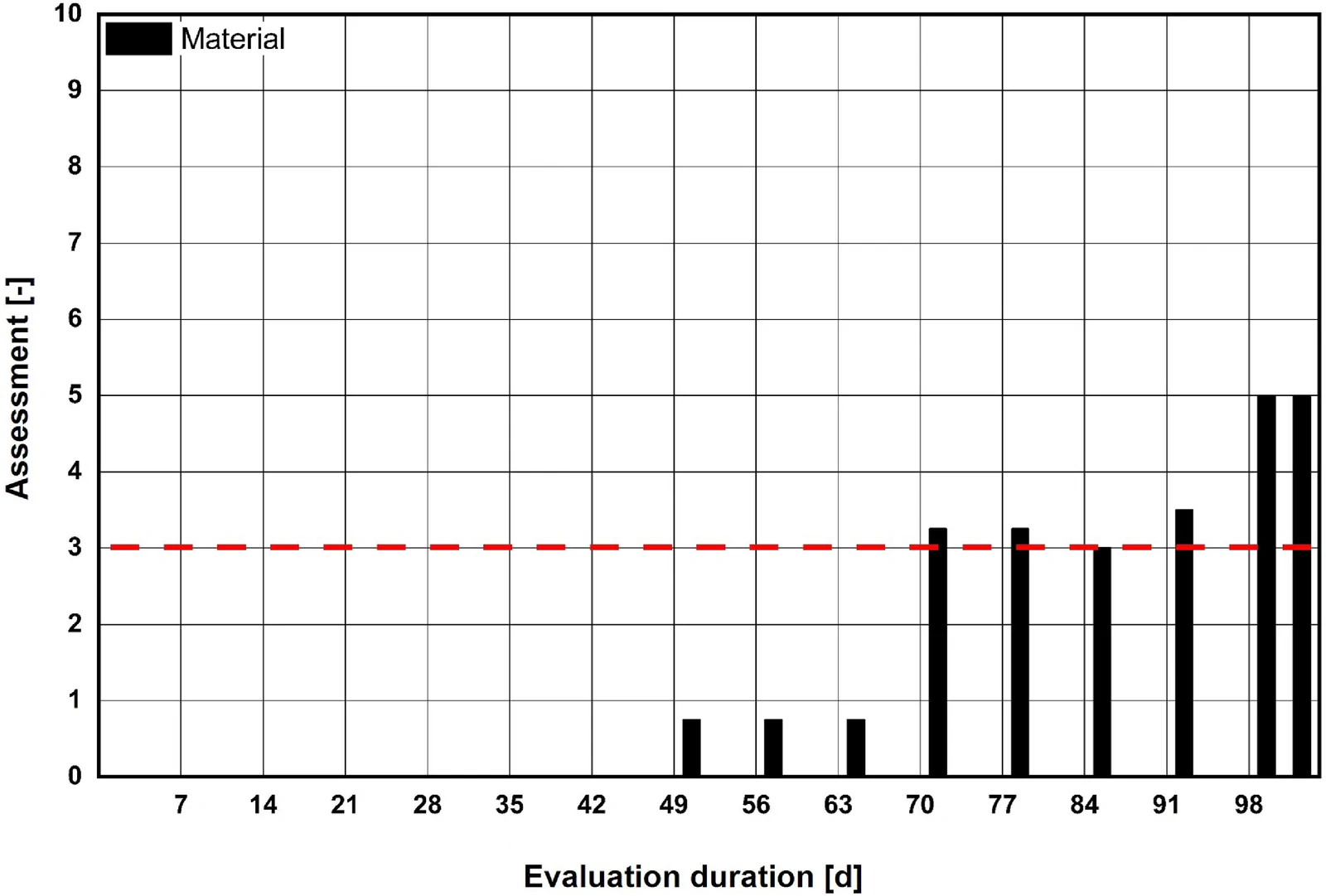
Fungal growth first appears in the 8th week of the test remaining at a low level. The fungal growth first exceeds the critical threshold of “3” after the 10th week of the test. In the last week the fungal growth reaches a level of “5”. The facade coating does not meet W3/A2 requirements.

**Fig. 11d:**
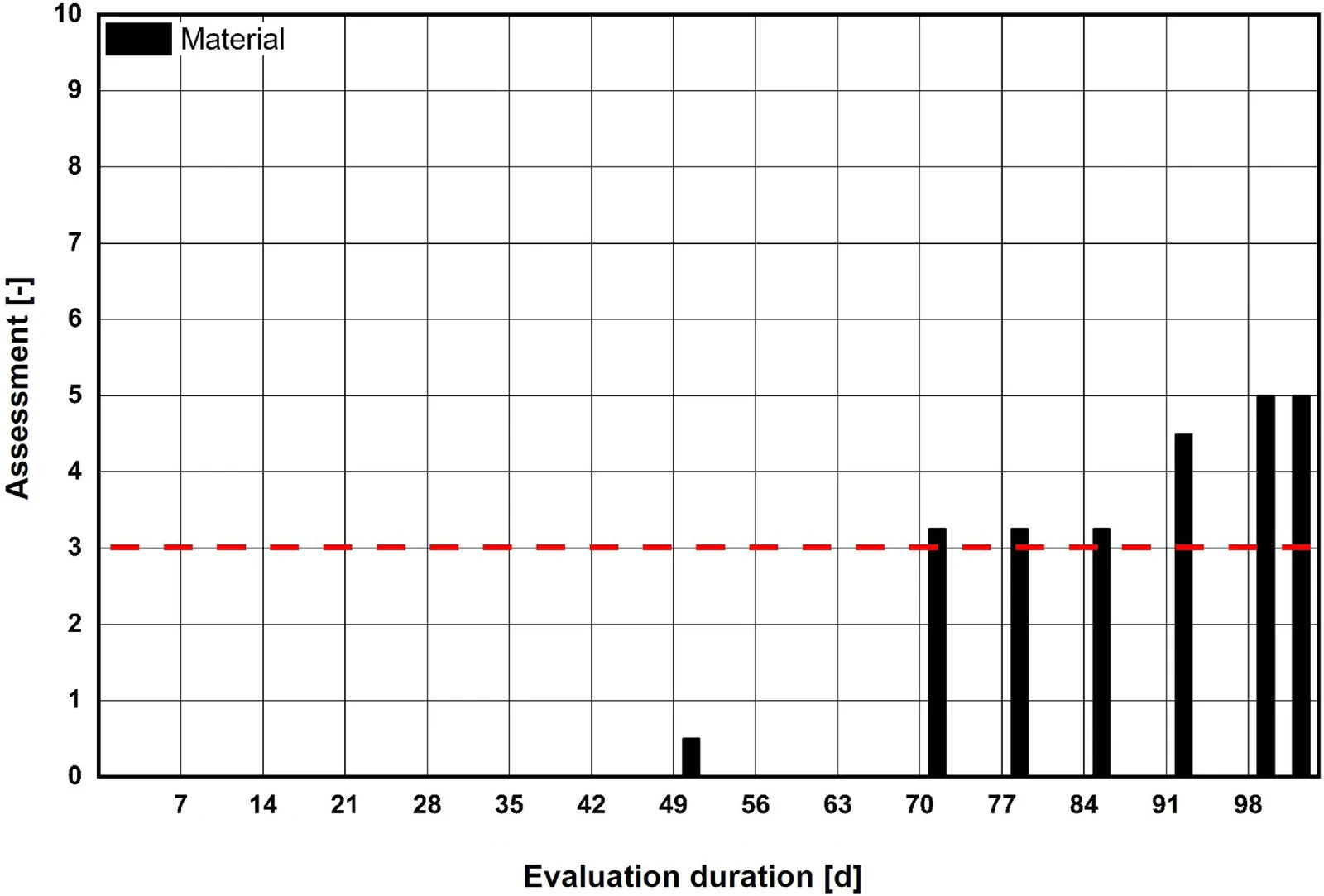
Fungal growth first appears in the 8th week of the test. By the 11th week of the test, the fungal growth has already exceeded the critical threshold of “3”. The facade coating does not demonstrate sufficient resistance to microbial growth (mold).

### Overall assessment

Taken together, the available evidence from repeated applications of the method in research and development projects [1,4], the clear discrimination between ETICS systems with different formulations and of different manufacturers, and the consistent behavior of positive and negative controls indicate that the rapid weathering procedure provides a reproducible and sensitive tool to compare the resistance of renders and facade paints against microbial growth and to support the development and quality assurance of biocide-free facade systems.

The outcomes of the first series of tests are summarized in Fig. 12. Starting in week 5 of the experiment (Fig. 13), the first trends regarding resistance to fungal growth become apparent. It is also evident that some materials show no fungal growth throughout the entire test period. This is particularly evident in ceramic veneers.

**Fig. 12:**
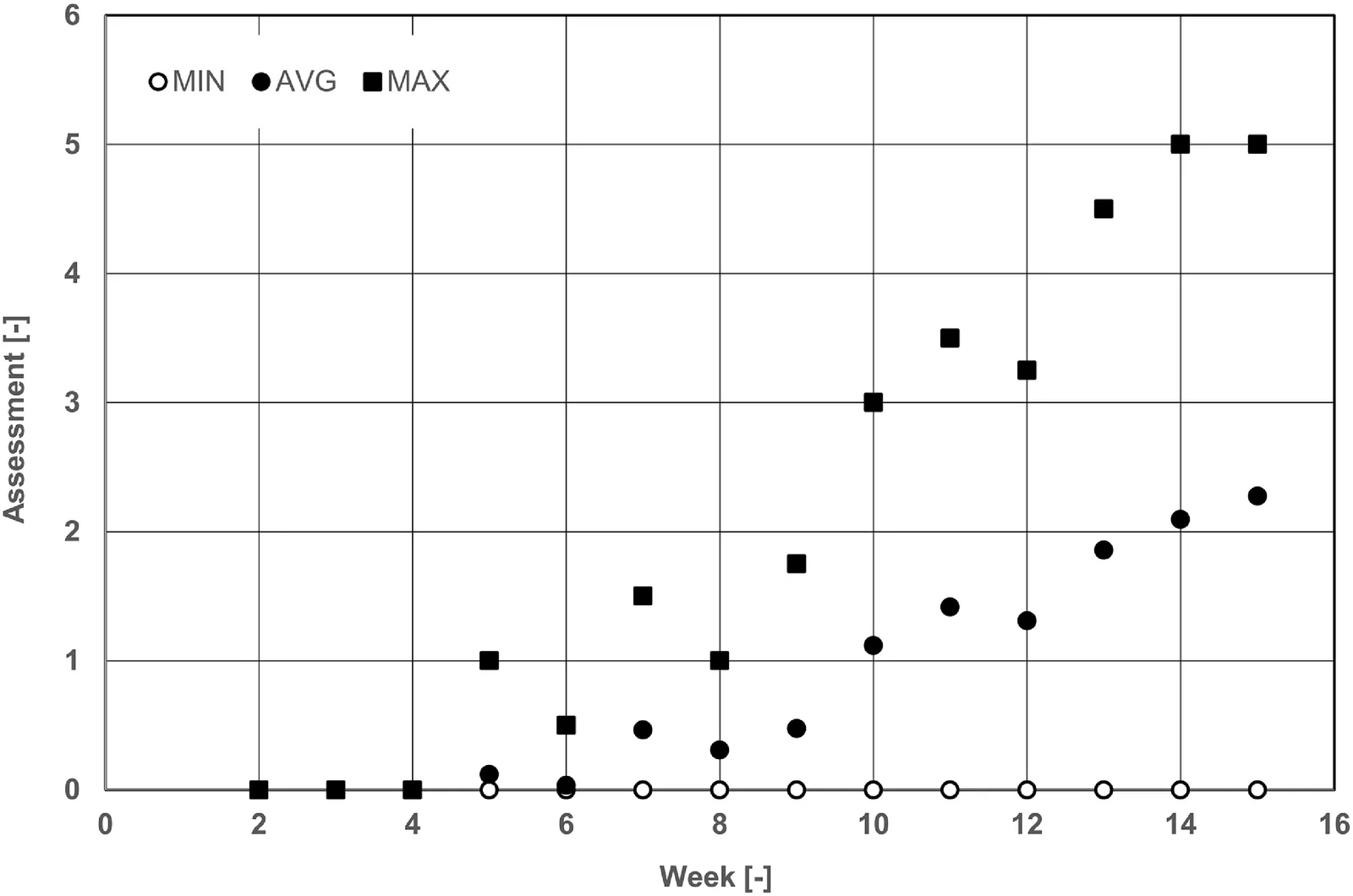
Compilation of test results. The table shows the minimum (MIN) and maximum (MAX) values determined in every test week, as well as the mean value (AVG).

**Fig. 13:**
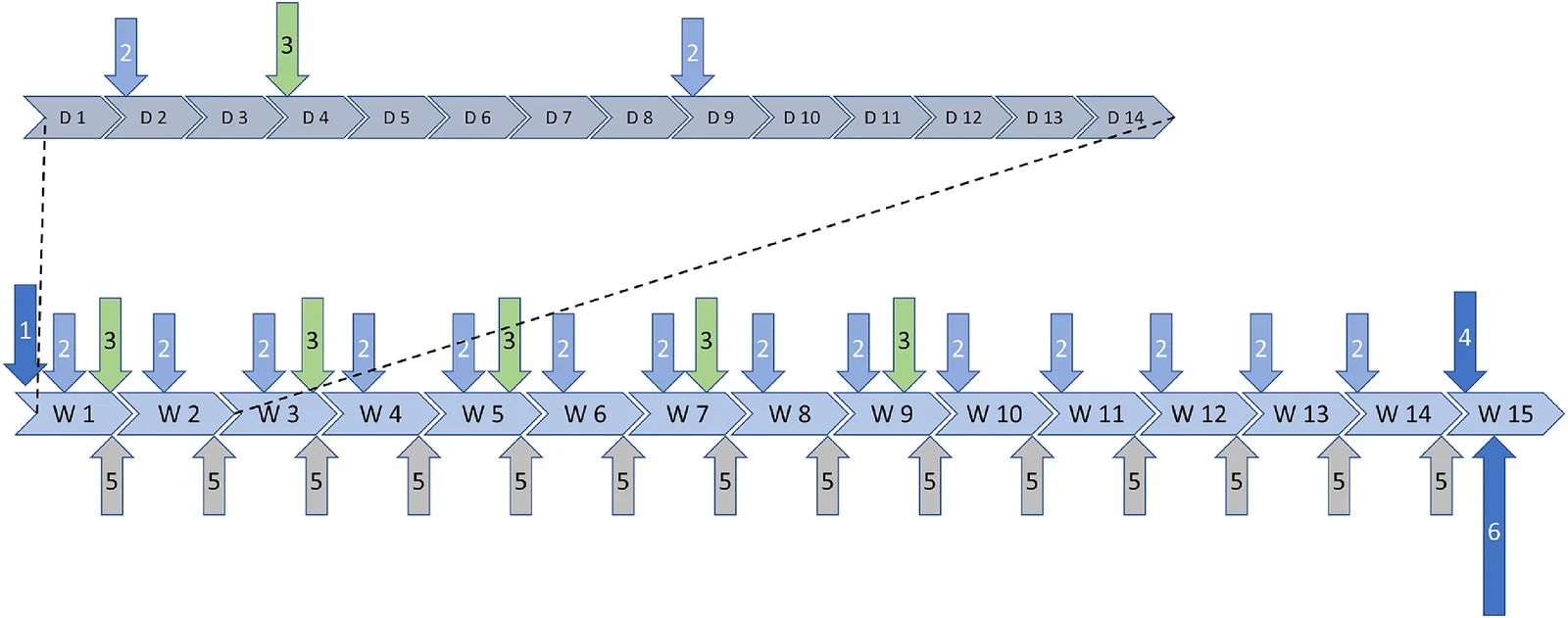
Timeline of the rapid weathering method; (1) Installation of test specimens and start of rapid weathering, (2) rain event, (3) inoculation with algae and mold suspension, (4) End of rapid weathering and removal of test specimens, (5) Assessment of microbial growth, (6) Examination of microbial growth on test specimens using a stereo microscope, final evaluation of microbial growth, photographic documentation W1 – W15: week number, D1 – D14: day number.

### Limitations

The method presented enables comparative studies on the resistance of plasters and paints to microbial growth. Ceramic claddings (brick slips with joints) were also successfully tested. The current design of the system only allows the investigation of components for vertical application. The applicability on coatings treated with biocides has not yet been tested. For testing materials that are installed horizontally or at a defined angle, the design of the rear panel and the positioning of the collection channels must be modified. In addition, it may also be necessary to modify the lighting to prevent shadows from affecting the test specimens.

The method presented with the parameters specified in Table 5 is suitable for the climatic conditions currently prevailing in Central Europe. Due to the free programmability of the climate parameters, other climates can also be realized with this system.

So far, no rapid weathering systems are commercially available that offer the capabilities of our system, particularly the temperature-controlled rear panel. It was therefore not possible to compare our system with those used in other laboratories.

Adaptation to different climates requires the availability of weather data spanning several years, which can be used to determine the diurnal cycle for the respective climate zone. A new climatic profile must be evaluated to determine whether it allows for a reliable assessment of resistance to microbial growth and provides sufficient discriminatory power. If different test organisms are required for another climate zone, different media and procedures may also be necessary for preparing the inoculation suspension.

## Ethics statements

No animal testing or human research was conducted. Data from social media was not used either.

## Related research article

N. Krueger, W.K. Hofbauer, A. Thiel, O. Ilvonen, Resilience of biocide-free ETICS to microbiological growth in an accelerated weathering test, Building and Environment 244 (2023) 110,737. https://doi.org/10.1016/j.buildenv.2023.110737

## CRediT author statement

**Christian R. Scherer**: Conceptualization; Writing - original draft; Writing – review & editing. **Nicole Krueger**: Investigation; Data curation; Writing – review & editing. **Wolfgang K. Hofbauer**: Methodology; Writing – review & editing. **Outi Ilvonen**: Writing – review & editing.

## Declaration of competing interest

The authors declare that they have no known competing financial interests or personal relationships that could have appeared to influence the work reported in this paper.

## Data Availability

The data that has been used is confidential.
